# Comparative purification of a *Salmonella* bacteriophage for protein profiling

**DOI:** 10.1007/s00253-026-13982-z

**Published:** 2026-07-28

**Authors:** Alinur Toleukhan, Akbope Abdykalyk, Aktoty Anarbekova, Azamat Abdimukhtar, Bekzat Yerzhigit, Nazym Syrym, Nadezhda Zinina, Sabira Alpysbayeva, Makhpal Sarmykova, Yeldos Serikbay, Kali Tileukhanov, Nurlan Kozhabergenov, Bekbolat Usserbayev, Yeraly Shayakhmetov, Kuandyk Zhugunissov, Sergazy Nurabayev, Andrey Shestakov, Bolat Yespembetov

**Affiliations:** 1 Research Institute for Biological Safety Problems, Gvardeyskiy, Kazakhstan; 2 Ulyanovsk State Agrarian University Named After P. A. Stolypin, Ulyanovsk, Russia

**Keywords:** *Salmonella* bacteriophage, Bacteriophage purification, SDS-PAGE profiling, Phage recovery, Ultrafiltration, Molecular safety screening

## Abstract

**Abstract:**

*Salmonella enterica* bacteriophages are increasingly studied for bacterial biocontrol and applied microbiology. However, reliable downstream characterization depends strongly on how the phage material is purified and concentrated. This study compared five preparation strategies for a *Salmonella* bacteriophage: sucrose density gradient centrifugation, cesium chloride gradient centrifugation, ultrafiltration followed by lyophilization, ultrafiltration followed by ammonium sulfate precipitation, and ultrafiltration followed by trichloroacetic acid precipitation. The preparations were assessed for infectious phage recovery, total protein concentration, SDS-PAGE protein profiles, an exploratory molecular weight-based comparison with a reference phage dataset, and targeted real-time PCR screening for selected virulence-associated determinants. Ultrafiltration followed by ammonium sulfate precipitation gave the highest infectious phage recovery among the infectivity-preserving workflows and produced a consistent eight-band SDS-PAGE profile. Trichloroacetic acid precipitation produced a similar banding pattern but was considered mainly a protein precipitation method because of its denaturing effect. Cesium chloride gradient centrifugation gave the most complex SDS-PAGE profile, while ultrafiltration followed by lyophilization gave the least resolved one. Several bands fell within the molecular-mass ranges reported for proteins of related *Salmonella* phages, but matching by molecular mass alone cannot identify the proteins, which would require LC-MS/MS. Targeted real-time PCR did not detect *sopE*, *sspH1*, *sseI*, or *sopE2*. Under the conditions tested, ultrafiltration followed by ammonium sulfate precipitation gave the highest infectious recovery and a consistent SDS-PAGE pattern; these results describe how the workflows behaved here rather than showing that one method is generally superior.

**Key points:**

• *Five workflows were compared for Salmonella phage purification.*

• *Ammonium sulfate precipitation gave the highest infectious recovery.*

• *Selected virulence-associated genes were not detected by qPCR.*

**Supplementary Information:**

The online version contains supplementary material available at https://doi.org/10.1007/s00253-026-13982-z.

## Introduction

*Salmonella enterica* remains an important foodborne pathogen affecting both humans and animals (Majowicz et al. 2010; Scallan et al. 2011). The spread of antimicrobial-resistant *Salmonella*, including isolates resistant to fluoroquinolones and extended-spectrum cephalosporins, has strengthened interest in alternative antibacterial strategies such as bacteriophage-based approaches (Medalla et al. 2017; Castro-Vargas et al. 2020; Hsu et al. 2023).

Reliable bacteriophage characterization depends strongly on the quality of the prepared phage material. Crude lysates may contain intact phage particles together with host-cell proteins, bacterial debris, nucleic acids, medium residues, and other lysis-derived components. These impurities can interfere with downstream protein-based analyses and complicate interpretation of electrophoretic profiles. Purification and concentration are therefore a critical methodological step, not a minor technical detail (Bourdin et al. 2014; Carroll-Portillo et al. 2021; Lapras et al. 2024).

Common bacteriophage preparation workflows include density-gradient centrifugation, ultrafiltration, precipitation, and combined approaches. These methods may differ in their ability to retain infectious particles, recover protein material, and generate clear and reproducible SDS-PAGE profiles (Bourdin et al. 2014; Carroll-Portillo et al. 2021; Kabwe et al. 2024). Comparative evaluation is therefore important when selecting a preparation strategy for downstream bacteriophage analysis.

SDS-PAGE remains a simple and accessible method for comparing bacteriophage protein profiles. Although it cannot definitively identify individual proteins, it allows assessment of apparent molecular-mass distribution, band reproducibility, and profile complexity across differently prepared samples. In parallel, targeted molecular screening can provide preliminary safety-related information, particularly because several *Salmonella* virulence-associated determinants have been linked to phage-mediated or prophage-related elements (Mirold et al. 1999; Ehrbar and Hardt 2005; Yates et al. 2024).

This study compared five purification and concentration approaches for a *Salmonella* bacteriophage and evaluated their suitability for infectious phage recovery, SDS-PAGE-based protein profiling, and preliminary molecular safety screening.

## Materials and methods

### Bacterial host strain and bacteriophage

A bacteriophage active against *Salmonella* spp. was used in this study. A susceptible *Salmonella enterica* strain served as the bacterial host for bacteriophage propagation and titration. The bacterial host strain and bacteriophage are maintained in the institutional collection of the corresponding author and are available for research purposes upon reasonable request. Bacterial cultivation and bacteriophage propagation were performed using GRM broth (pancreatic hydrolysate of fish meal 18 g/L, sodium chloride 2 g/L) and GRM agar (pancreatic hydrolysate of fish meal 12 g/L, enzymatic peptone 12 g/L, sodium chloride 6 g/L, microbiological agar 10 g/L) (Obolensk, Russia). GRM is a rich, non-selective nutrient medium standardized for the cultivation of *Enterobacteriaceae*, including *Salmonella* and reliably supported high-density host growth and high phage titers in our laboratory.

The initial bacteriophage suspension had an approximate titer of 1 × 10^9^ PFU/mL, as determined by the Gratia double-layer agar method, and was used as the starting material for all purification and concentration procedures.

### Experimental design

Five workflows were compared for preparation of the *Salmonella* bacteriophage for downstream protein profiling and preliminary molecular safety screening: sucrose density-gradient centrifugation, cesium chloride gradient centrifugation, ultrafiltration followed by lyophilization, ultrafiltration followed by ammonium sulfate precipitation, and ultrafiltration followed by trichloroacetic acid precipitation.

Each preparation was evaluated for infectious phage recovery, total protein concentration, SDS-PAGE protein profiling, and densitometric assessment of apparent molecular masses. Bacteriophage recovery and protein concentration were assessed using technical replicates derived from the same initial bacteriophage preparation. Starting volumes differed between the gradient-based workflows (3–4 mL) and the ultrafiltration-based workflows (100 mL) because density-gradient loading is inherently volume-limited, whereas ultrafiltration is designed to process and concentrate larger volumes. This difference was partially accounted for by expressing recovery as the ratio of total infectious particles (final total PFU/initial total PFU), which reflects the proportion of phage retained rather than the concentration factor. For comparison across methods, all final preparations were adjusted to 1.0 mL after purification or concentration.

In addition, DNA extracted from the common bacteriophage stock was subjected to targeted real-time PCR as a separate preliminary molecular safety screening step.

**Fig. 1:**
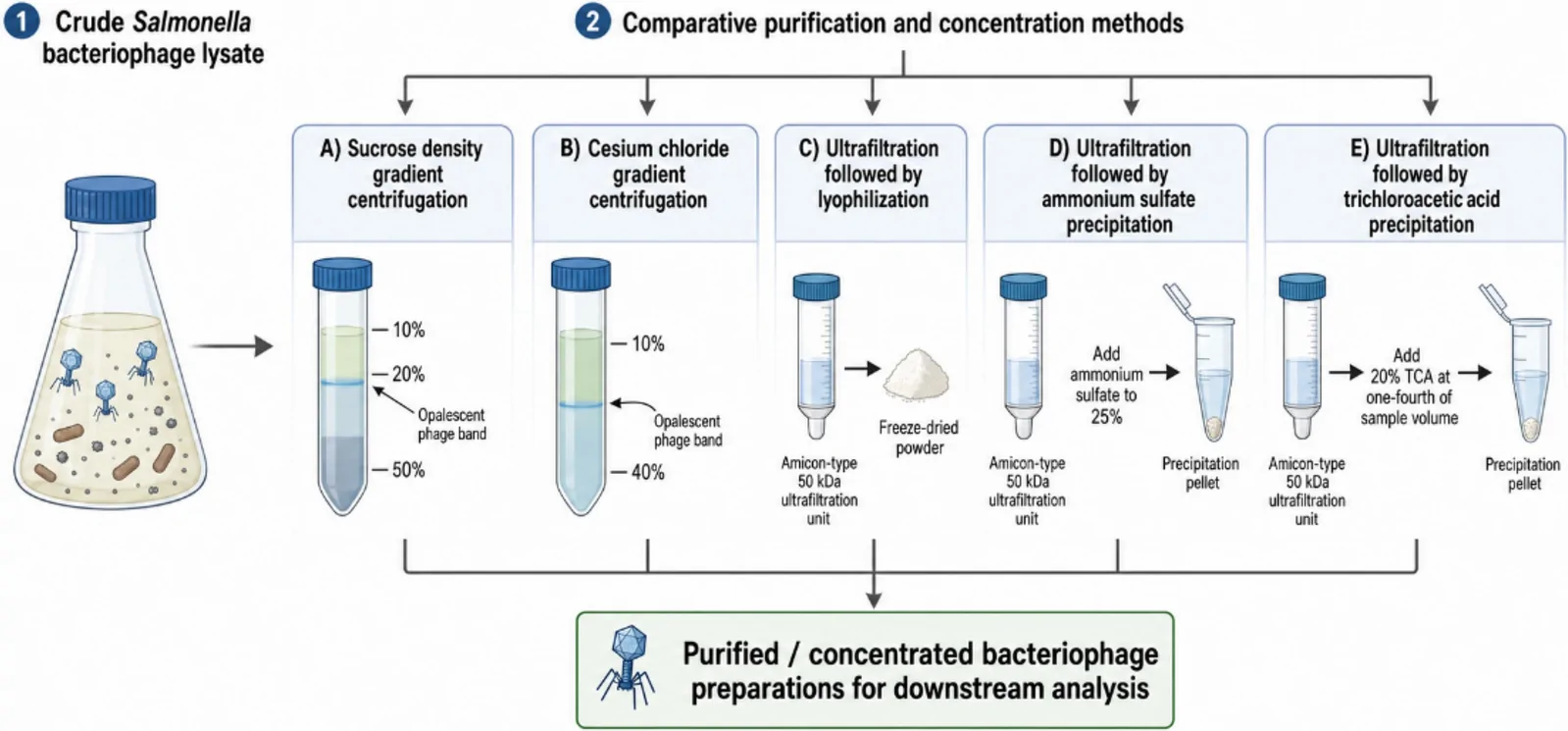
Workflow of the five purification and concentration procedures applied to the crude *Salmonella* bacteriophage lysate before downstream analysis

The most consistent SDS-PAGE profile was selected for preliminary molecular weight-based comparison with annotated proteins of related *Salmonella* bacteriophages. This comparison was interpreted as a descriptive structural comparison rather than definitive protein identification. A schematic overview of the study design and purification procedures is presented in Fig. 1.

### Clarification of the crude bacteriophage lysate

Before purification, the bacteriophage suspension was clarified to remove large bacterial debris and particulate material. For ultrafiltration-based procedures, the suspension was centrifuged at 4000 rpm (3000 × g) for 20 min using an Eppendorf 5804 centrifuge (Eppendorf, Germany), and the clarified supernatant was used for further processing. For gradient-based methods, aliquots of the initial suspension were layered directly onto the corresponding density gradients.

### Purification and concentration procedures

#### Sucrose density gradient centrifugation

Sucrose-based centrifugation is commonly used to concentrate and partially purify viral particles, including bacteriophages (Bourdin et al. 2014; Petrova et al. 2020). In this study, a discontinuous sucrose gradient consisting of 10%, 20%, and 50% (w/v) sucrose was used.

In a 12-mL centrifuge tube, 3 mL of 50% (w/v) sucrose was added as the bottom layer. The 20% and 10% (w/v) sucrose solutions were then carefully layered sequentially above it. A 3-mL aliquot of bacteriophage suspension with an approximate titer of 1 × 10^9^ PFU/mL, as determined by the Gratia double-layer agar method, was layered onto the top of the gradient.

The tubes were centrifuged at 14,000 rpm (20,000 × g) for 10 h. After centrifugation, a visible opalescent band was observed at the interface between the 10% and 20% sucrose layers. This band was collected as the phage-containing fraction and adjusted to a final volume of 1.0 mL with 0.01 M Tris-HCl buffer.

#### Cesium chloride gradient centrifugation

Cesium chloride gradient purification was performed using 10% and 40% (w/v) CsCl solutions prepared in 0.01 M Tris-HCl buffer. An 8-mL linear CsCl gradient was prepared in a 12-mL ultracentrifuge tube using 4 mL of each solution. A 4-mL aliquot of bacteriophage suspension with an approximate titer of 1 × 10^9^ PFU/mL, as determined by the Gratia double-layer agar method, was carefully layered onto the gradient.

The gradients were centrifuged at 14,000 rpm (20,000 × g) for 20 h. After centrifugation, the visible opalescent phage-containing fraction was collected and adjusted to a final volume of 1.0 mL with 0.01 M Tris-HCl buffer.

#### Ultrafiltration followed by lyophilization

The bacteriophage suspension was clarified by centrifugation at 4000 rpm (3000 × g) for 20 min using an Eppendorf 5804 centrifuge. A total of 100 mL clarified suspension was concentrated for 2 h using Amicon Ultra-15 centrifugal filter units with a 50-kDa molecular weight cut-off (Merck Millipore, Germany).

The concentrated preparation was lyophilized at −80 °C using an LL-3000 lyophilizer and reconstituted to 1.0 mL in 0.01 M Tris–HCl.

#### Ultrafiltration followed by ammonium sulfate precipitation

The bacteriophage suspension was concentrated using Amicon Ultra-15 centrifugal filter units with a 50-kDa molecular-weight cut-off. Solid ammonium sulfate was added to the ultrafiltration concentrate to a final concentration of 25% (w/v). This concentration was used as an empirically selected working condition for evaluating the combined ultrafiltration–ammonium sulfate precipitation workflow. No preliminary concentration-response optimization was performed. Therefore, the 25% (w/v) condition should be regarded as an operational parameter specific to the present study rather than an optimal or generally applicable precipitation concentration.

The mixture was gently agitated until the ammonium sulfate had completely dissolved and was then incubated for 2 h at 2 °C. Samples were centrifuged at 13,000 rpm (16,000 × g) for 30 min using a Microspin FV-2400 microcentrifuge (BioSan, Latvia). The precipitated material was collected and resuspended to a final volume of 1.0 mL in 0.01 M Tris–HCl buffer.

#### Ultrafiltration followed by trichloroacetic acid precipitation

The bacteriophage suspension was concentrated using Amicon Ultra-15 centrifugal filter units with a 50-kDa molecular weight cut-off. A 20% trichloroacetic acid solution was added at one-fourth of the sample volume.

The mixture was gently agitated and centrifuged at 13,000 rpm (16,000 × g) for 30 min using a Microspin FV-2400 microcentrifuge. The precipitated material was collected and resuspended to a final volume of 1.0 mL in 0.01 M Tris-HCl buffer. Because trichloroacetic acid causes irreversible protein precipitation and may reduce bacteriophage infectivity, this workflow was interpreted primarily as a protein precipitation method.

#### Determination of bacteriophage recovery

Bacteriophage recovery was determined using the Gratia double-layer agar plaque assay with the susceptible *Salmonella enterica* host strain. Phage titers were measured before and after each purification procedure using technical replicate measurements.

Serial tenfold dilutions were prepared in sterile buffer. Aliquots of each dilution were mixed with host bacterial culture and molten soft agar, then overlaid onto GRM agar plates. After incubation under standard bacterial growth conditions, plaques were counted on plates containing countable plaque numbers, and titers were expressed as PFU/mL.

$$Recovery\;\left(\%\right)=\frac{Final\;total\;PFU}{Initial\;total\;PFU}\times100$$

#### Determination of total protein concentration

Total protein concentration was determined for the crude bacteriophage lysate and purified preparations using the Pierce™ BCA Protein Assay Kit (Thermo Fisher Scientific, Waltham, MA, USA) according to the manufacturer’s instructions. Measurements were performed in technical replicates.

Protein concentration was expressed as mg/mL. The final protein amount was calculated from the measured protein concentration and the standardized final sample volume of 1.0 mL.

#### SDS-PAGE analysis

Protein samples were prepared according to a modified Laemmli protocol (Laemmli 1970). Briefly, 50 µL of bacteriophage suspension or purified preparation was mixed with 200 µL of 1% sodium dodecyl sulfate preheated to 95 °C. The mixture was vortexed and subjected to ultrasonic disruption using a Soniprep 150 ultrasonic homogenizer (MSE, UK) at 60 W and 20 kHz for 30 s.

Samples were heated at 95 °C for 5 min and cooled to approximately 20 °C. Then, 250 µL of 2 × Laemmli sample buffer was added, and samples were incubated at room temperature for 20 min. Preparations were centrifuged at 13,000 rpm (16,000 × g) for 30 min using a Microspin FV-2400 microcentrifuge, and the supernatants were used for electrophoresis.

The 2 × Laemmli sample buffer contained 62.5 mM Tris-HCl, pH 6.8, 2% sodium dodecyl sulfate, 25% glycerol, 0.01% bromophenol blue, and 5% β-mercaptoethanol. Protein separation was performed using Criterion TGX 8–16% polyacrylamide gels (Bio-Rad, USA) in a Mini-PROTEAN Tetra vertical electrophoresis system connected to a PowerPac Basic power supply (Bio-Rad, USA). A 3.5–245 kDa protein molecular-weight marker (Abcam, UK) was used to estimate apparent molecular masses.

Electrophoresis was performed for 45–60 min. Gels were stained with Coomassie Brilliant Blue R-250 for 2 h at 60 °C and destained with 10% acetic acid for 2 h at 60 °C until bands were clearly visible.

#### Densitometric analysis and in silico protein matching

Stained gels were documented and analyzed using Bio-Vizion software (Vilber, France). Apparent molecular masses were estimated from the relative migration of the detected bands compared with the molecular weight marker. These estimates were used for descriptive comparison of the electrophoretic profiles and not for definitive protein identification. Protein profiles were compared according to the number of visible bands, molecular mass distribution, band recurrence across methods, and profile clarity.

The selected SDS-PAGE profile was compared with annotated proteins of related *Salmonella* bacteriophages available in the NCBI Protein database. Matching was based only on approximate molecular mass correspondence and was interpreted as preliminary structural comparison.

#### Real-time PCR screening of selected virulence-associated determinants

Targeted real-time PCR screening was performed for selected *Salmonella* virulence-associated determinants, including *sopE*, *sspH1*, *sseI*, and *sopE2*. Reference nucleotide sequences were retrieved from the NCBI database and checked by BLAST-based comparison before primer and probe design.

DNA was extracted using the PROBA-RAPID DNA extraction kit (DNA-Technology, Russia) according to the manufacturer’s instructions. Real-time PCR was performed using a DTprime 4M1 real-time PCR detection system (DNA-Technology, Russia).

Each 25-µL reaction contained 12.5 µL reaction mixture, 1 µL primer mix, 1 µL fluorescent probe, 5 µL DNA template, and nuclease-free water to the final volume. The amplification protocol consisted of initial denaturation/enzyme activation at 95 °C for 5 min, followed by 5 preliminary cycles of 95 °C for 10 s and 60 °C for 30 s, and 40 amplification cycles of 95 °C for 10 s and 60 °C for 20 s. Fluorescence acquisition was performed during the 60 °C step of the main cycles.

This analysis was interpreted as preliminary molecular safety screening and not as a substitute for whole-genome sequencing.

### Statistical and comparative analysis

The study was designed as a comparative methodological evaluation of bacteriophage purification and concentration workflows. Infectious phage recovery, protein concentration, final protein amount, and SDS-PAGE profile characteristics were summarized descriptively.

Values obtained from technical replicate measurements were expressed as mean ± standard deviation where applicable. No inferential statistical testing was applied because the study was designed as a comparative methodological evaluation.

## Results

### Comparative recovery of bacteriophage preparations

The initial bacteriophage suspension had an approximate titer of 1 × 10^9^ PFU/mL. Table 1 presents sample volumes and phage titers separately from total infectious yield. Recovery was calculated from the initial and final total PFU values rather than from PFU/mL alone.

**Table 1:** Infectious bacteriophage recovery after different purification and concentration procedures

Method	Sample volume		Phage concentration		Total infectious yield		
	Initial volume, mL	Final volume, mL	Initial titer, PFU/mL	Final titer, PFU/mL	Initial total PFU	Final total PFU	Recovery, %
Sucrose density gradient	3.0	1.0	1 × 10^9^	7.8 × 10^8^	3.0 × 10^9^	7.8 × 10^8^	26.0
Cesium chloride gradient	4.0	1.0	1 × 10^9^	6.5 × 10^8^	4.0 × 10^9^	6.5 × 10^8^	16.3
Ultrafiltration + lyophilization	100.0	1.0	1 × 10^9^	2.4 × 10^10^	1.0 × 10^11^	2.4 × 10^10^	24.0
Ultrafiltration + ammonium sulfate	100.0	1.0	1 × 10^9^	3.8 × 10^10^	1.0 × 10^11^	3.8 × 10^10^	38.0
Ultrafiltration + TCA	100.0	1.0	1 × 10^9^	NA	1.0 × 10^11^	NA	NA

Recovery was calculated as final total PFU divided by initial total PFU × 100. TCA precipitation was used primarily for protein precipitation and was not evaluated for infectious phage recovery

*NA* not applicable

Among the infectivity-preserving workflows, ultrafiltration followed by ammonium sulfate precipitation showed the highest recovery (38.0%). Sucrose density gradient centrifugation and ultrafiltration followed by lyophilization showed comparable recoveries of 26.0% and 24.0%, respectively, whereas cesium chloride gradient centrifugation showed the lowest recovery (16.3%). Trichloroacetic acid precipitation was not evaluated for infectious phage recovery because this workflow was used primarily for protein precipitation and may compromise phage infectivity.

### Total protein concentration and final protein amount

Total protein concentration differed among the tested preparation methods. The crude bacteriophage lysate contained 1.82 ± 0.14 mg/mL of total protein. Among purified or concentrated preparations, the highest protein concentrations were observed after trichloroacetic acid precipitation and ammonium sulfate precipitation, with 0.81 ± 0.08 and 0.76 ± 0.07 mg/mL, respectively (Table 2). Cesium chloride gradient centrifugation yielded 0.71 ± 0.06 mg/mL, whereas sucrose density gradient centrifugation and ultrafiltration followed by lyophilization yielded lower protein concentrations of 0.46 ± 0.05 and 0.38 ± 0.04 mg/mL, respectively.

**Table 2:** Total protein concentration and final protein amount in bacteriophage preparations

Method	Protein concentration, mg/mL	Final volume, mL	Final protein amount, mg
Crude bacteriophage lysate	1.82 ± 0.14	1.0	1.82
Sucrose density gradient	0.46 ± 0.05	1.0	0.46
Cesium chloride gradient	0.71 ± 0.06	1.0	0.71
Ultrafiltration + lyophilization	0.38 ± 0.04	1.0	0.38
Ultrafiltration + ammonium sulfate	0.76 ± 0.07	1.0	0.76
Ultrafiltration + trichloroacetic acid	0.81 ± 0.08	1.0	0.81

Protein concentration values are presented as mean ± standard deviation of technical replicate measurements. Because all final preparations were standardized to 1.0 mL, the final protein amount corresponded numerically to the measured protein concentration

### SDS-PAGE-based comparison of crude and purified preparations

**Fig. 2:**
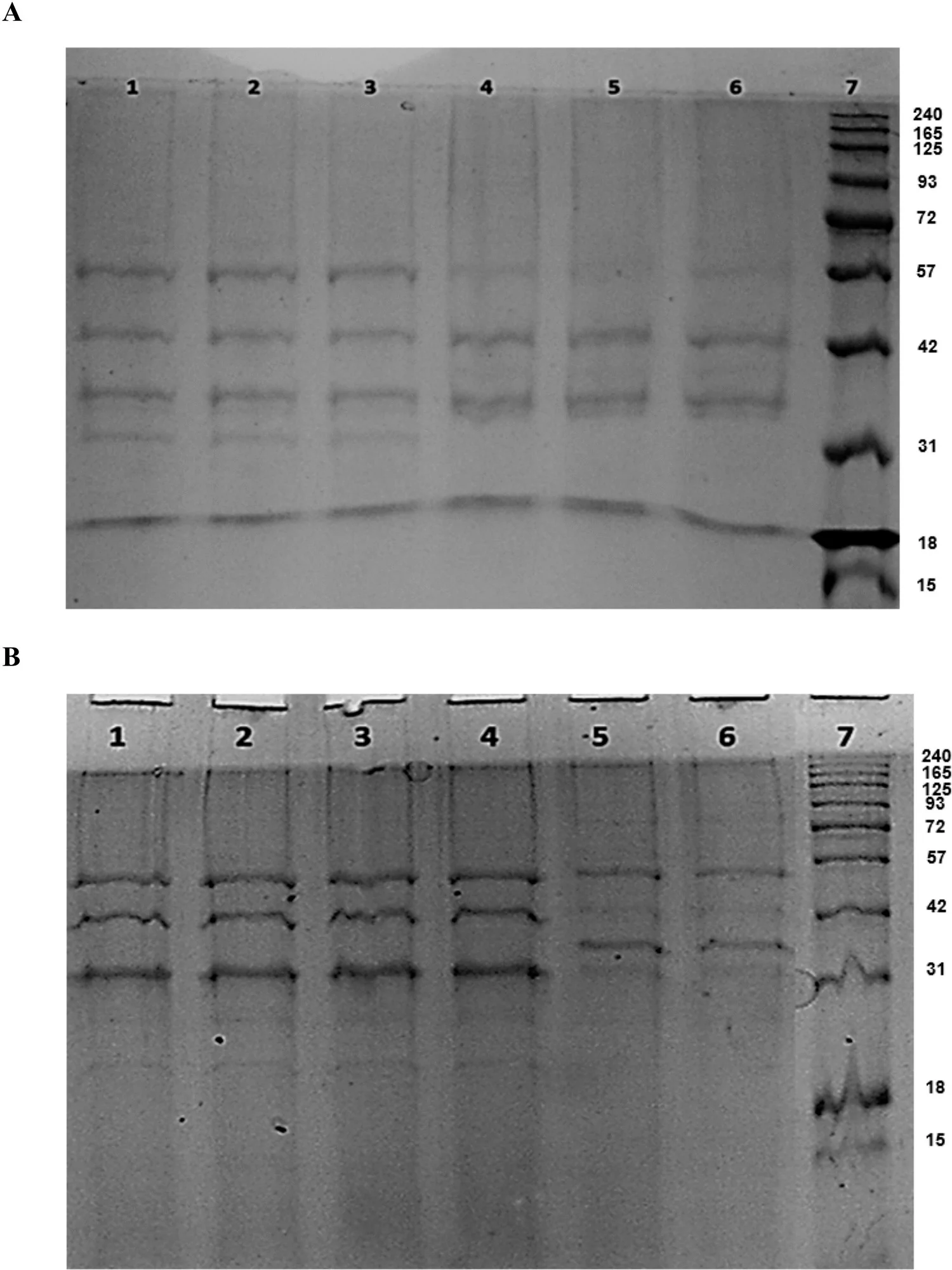
Representative SDS-PAGE profiles of *Salmonella* bacteriophage preparations obtained using different purification and concentration workflows. **A** Lanes 1–3, sucrose density-gradient centrifugation; lanes 4–6, ultrafiltration followed by lyophilization; lane 7, molecular-weight marker. **B** Lanes 1–2, ultrafiltration followed by ammonium sulfate precipitation; lanes 3–4, ultrafiltration followed by trichloroacetic acid precipitation; lanes 5–6, cesium chloride density-gradient centrifugation; lane 7, molecular-weight marker

The apparent SDS-PAGE profile varied among the preparation workflows. Compared with the crude bacteriophage lysate, the purified and concentrated preparations showed visually more discrete electrophoretic banding patterns, although the number and distribution of visible bands differed among workflows (Table 3; Fig. 2; Fig. S1; Fig. S2).

**Table 3:** SDS-PAGE protein profiles of *Salmonella* bacteriophage preparations obtained after different purification and concentration procedures

Method	Detected apparent protein bands, kDa	Number of bands	Interpretation
Crude bacteriophage lysate	Multiple/background bands	-	Complex background profile
Sucrose density gradient	66.5, 57.8, 42.6, 37.8, 36.5, 33.3	6	Intermediate profile
Cesium chloride gradient	89.7, 74.1, 58.4, 52.5, 41.8, 36.1, 32.7, 25.5, 21.5, 17.8	10	Broadest detectable profile
Ultrafiltration + lyophilization	58.5, 42.6, 39.7, 37.0, 35.8	5	Limited detectable profile
Ultrafiltration + ammonium sulfate	87.0, 57.0, 50.3, 40.8, 32.1, 25.2, 21.1, 17.8	8	Consistent working profile
Ultrafiltration + trichloroacetic acid	88.6, 57.9, 50.3, 41.2, 32.3, 25.4, 21.2, 17.8	8	Consistent protein-precipitated profile

Cesium chloride gradient centrifugation produced the broadest detectable profile, with ten visible bands ranging from approximately 17.8 to 89.7 kDa. Sucrose density gradient centrifugation produced six detectable bands, whereas ultrafiltration followed by lyophilization produced the most limited profile with five visible bands. Ultrafiltration followed by ammonium sulfate precipitation and trichloroacetic acid precipitation produced highly similar eight-band profiles, including recurring bands in the approximate regions of 87–89, 57–58, 50, 41, 32, 25, 21, and 18 kDa.

The eight-band profile obtained after ultrafiltration followed by ammonium sulfate precipitation was selected for downstream molecular weight-based interpretation because it combined consistent band distribution with preservation of infectious phage recovery.

### Preliminary molecular weight-based structural protein matching

The working SDS-PAGE profile obtained after ultrafiltration followed by ammonium sulfate precipitation was compared with annotated proteins of related *Salmonella* bacteriophages available in the NCBI Protein database. The closest reference profile used for comparison was *Salmonella* phage ST0223.

Several observed bands showed approximate molecular mass correspondence with proteins annotated in the selected ST0223 reference dataset. Bands in the regions of 17.8, 21.1, 25.2, 32.1, 57.0 and 87.0 kDa showed the closest correspondence with annotated proteins in the selected reference set (Table 4). The 40.8 kDa band showed the nearest correspondence to a predicted 42.996 kDa protein, whereas the 50.3 kDa band did not show a close one-to-one match within the selected ST0223 protein list.

**Table 4:** Exploratory comparison of observed SDS-PAGE bands with predicted protein masses in the *Salmonella* phage ST0223 reference dataset

Observed SDS-PAGE band, kDa	Closest predicted ST0223 protein mass, Da	Closest molecular-mass correspondence in the selected reference dataset
17.8	17,235/17,498/17,937	Endonuclease/hypothetical protein/internal virion protein
21.1	21,444	Tail protein
25.2	25,547	ssDNA binding and annealing protein
32.1	31,820/33,084	Head assembly protein/exonuclease
40.8	42,996	DNA ligase or related predicted protein
50.3	No close single match in the selected ST0223 list	No close molecular-mass correspondence in the selected reference dataset
57.0	57,168	Head-tail adaptor
87.0	85,257/86,279	Internal virion protein/tail protein

Matching bands by apparent molecular mass alone is not the same as identifying the proteins. The proteins listed here simply show what is present in the reference phage, and some of them, such as the endonuclease, exonuclease, and DNA ligase, are replication-associated proteins that are unlikely to be part of the mature virion. Identifying the actual proteins in these bands would require sequencing the studied phage and analysing the bands by LC-MS/MS.

### Screening for selected virulence-associated determinants

Targeted real-time PCR screening was performed for selected *Salmonella* virulence-associated determinants, including *sopE, sspH1*, *sseI*, and *sopE2* (Table 5). These targets were included because they have been reported in association with phage-mediated or prophage-related elements in *Salmonella*. This screening was performed as a separate preliminary molecular safety assessment of the common bacteriophage stock rather than as an endpoint for comparing the purification workflows. Because all preparations were derived from the same stock, differences in the selected genetic targets between workflows were not expected. No target-specific amplification was detected for any of the four selected genes under the assay conditions used. Specific fragments corresponding to *sopE*, *sspH1*, *sseI*, and *sopE2* were therefore not detected in the studied bacteriophage preparation.

**Table 5:** Real-time PCR screening of selected virulence-associated determinants

Target gene	Reason for inclusion	Real-time PCR result
*sopE*	Type III secretion effector; reported in association with phage/prophage-related elements in *Salmonella*	Not detected
*sspH1*	*Salmonella* effector associated with virulence	Not detected
*sseI*	*Salmonella* effector associated with virulence	Not detected
*sopE2*	Type III secretion effector associated with *Salmonella* pathogenicity	Not detected

No target-specific amplification was detected for the four selected determinants under the assay conditions used. These results provide only limited, targeted information and do not demonstrate the genome-wide absence of virulence-associated genes. Comprehensive safety assessment would require whole-genome sequencing and genome annotation.

## Discussion

This study shows that purification and concentration strategy has a clear effect on the quality and interpretability of *Salmonella* bacteriophage preparations. Although all workflows were applied to the same initial phage material, they differed in infectious recovery, final protein amount, and electrophoretic banding pattern. This indicates that phage purification is not only a preparative step, but also an important methodological factor that can influence downstream characterization (Lapras et al. 2024; Petrova et al. 2020).

Cesium chloride gradient centrifugation produced the broadest SDS-PAGE profile, with ten visible bands. This is consistent with the established use of CsCl gradients for concentrating and separating viral particles for structural and molecular analyses (Joklik 1962; Bourdin et al. 2014). However, because no matched host-only control or LC-MS/MS confirmation was available, the origin of the additional bands could not be determined. We therefore interpreted the broader pattern only as greater electrophoretic profile complexity, rather than as evidence of higher purity or a larger number of phage structural proteins. CsCl purification also resulted in the lowest infectious recovery among the infectivity-preserving methods, in line with previous reports showing that purification procedures can reduce phage recovery or activity depending on the phage and processing conditions (Carroll-Portillo et al. 2021). Under the conditions tested, ultrafiltration followed by ammonium sulfate precipitation gave the highest calculated infectious recovery and a consistent eight-band SDS-PAGE profile. In contrast, trichloroacetic acid precipitation produced a similar banding pattern, but its denaturing effect makes it more appropriate for protein recovery than for preparations that need active phage particles. Because the workflows differed in starting scale and only technical replicates were available, this result should not be read as evidence that ammonium sulfate precipitation is generally superior. Ultrafiltration followed by lyophilization retained infectious particles but produced the most limited banding pattern. Lyophilization may still be useful for storage or formulation, but its performance depends on phage morphology, buffer composition, stabilizers, freezing parameters, and reconstitution conditions. In this study, it was less informative for SDS-PAGE-based characterization.

Several of the observed bands fell within the molecular-mass ranges reported for structural and virion-associated proteins of related *Salmonella* bacteriophages, including major head, tail, and internal virion proteins (Adriaenssens et al. 2012; Kosznik-Kwaśnicka et al. 2020; Boulanger 2009). However, matching by molecular mass alone is not enough to identify proteins, especially when the comparison is made against a different reference phage rather than the sequenced genome of the studied phage. The reference set also included non-structural, replication-associated proteins, so the correspondence should be read only as a rough indication of the mass range involved. Confirming which proteins are actually present would require sequencing the studied phage and analysing the bands by LC-MS/MS (Adriaenssens et al. 2012).

Targeted real-time PCR did not detect *sopE*, *sspH1*, *sseI*, or *sopE2* in the studied bacteriophage preparation. These targets were selected because several *Salmonella* virulence-associated determinants have been reported in association with phage-mediated or prophage-related elements (Ehrbar and Hardt 2005; Mirold et al. 1999). This screening was intended as a preliminary molecular safety assessment rather than as a comparative endpoint between purification methods because all preparations were derived from the same phage stock. The absence of these selected targets provides only limited molecular safety information and should not be interpreted as evidence of genome-wide safety. A comprehensive assessment would require whole-genome sequencing and annotation of virulence-associated, lysogeny-related, and antimicrobial resistance genes, as recommended in current discussions on phage suitability (Cook and Hynes 2025).

This study has several limitations. The experiments were performed using technical replicate preparations derived from the same initial bacteriophage material; future work should incorporate biological replicates from independent lysates to strengthen quantitative comparisons. SDS-PAGE provided only approximate molecular-mass information and did not allow definitive protein identification, which would require LC-MS/MS or another high-resolution proteomic method. A phage-free preparation of the uninfected host was not processed in parallel through the purification workflows. Therefore, the SDS-PAGE data could not distinguish phage-associated proteins from co-purified host-derived proteins or other background material. Future studies should include a matched phage-free host lysate processed through each workflow under the same conditions. The PCR screening was limited to four selected virulence-associated determinants and did not replace genome-wide analysis; whole-genome sequencing with annotation of virulence, lysogeny, and antimicrobial resistance genes would provide a more complete safety assessment. In addition, the performance of each purification workflow may vary depending on the phage, lysate composition, buffer system, and intended downstream application and validation using additional bacteriophages would help define the generalisability of these findings. The differing starting volumes may also have affected precipitation efficiency and plaque-assay measurements despite calculation of recovery from initial and final total PFU. Future comparative studies should therefore use matched starting volumes and matched initial total PFU whenever technically feasible, include independent biological preparations, and perform plaque assays in parallel using the same host culture, dilution scheme, agar batch, incubation conditions, and plaque-counting criteria.

Under the conditions tested, ultrafiltration followed by ammonium sulfate precipitation gave the highest calculated infectious phage recovery, while also providing enough protein for analysis and a consistent SDS-PAGE pattern. More broadly, the results show that the purification step can directly affect both phage recovery and the electrophoretic profile obtained from the same starting material. This means that the choice of workflow should depend on the main purpose of the study, whether that is preserving infectivity, recovering protein, comparing SDS-PAGE profiles, or preparing phage material for further formulation and applied research. At the same time, these findings are specific to the phage and experimental conditions used here and should not be interpreted as proof that one method is universally superior (Kutateladze and Adamia 2010; Makalatia et al. 2021).

## Supplementary Information

Below is the link to the electronic supplementary material.

ESM1 (DOCX 1.75 MB)

## Data Availability

No datasets were generated or analysed during the current study.
