# Supplementary material for: Comparative purification of a Salmonella bacteriophage for protein profiling
Source: Appl Microbiol Biotechnol. 2026 Jul 28;110:318. doi: 10.1007/s00253-026-13982-z (PMC13694477; doi:10.1007/s00253-026-13982-z)
Supplement: Supplementary file 1 [file 253_2026_13982_MOESM1_ESM.docx]

A

B

C

D

**Supplementary Fig. S1.** Supplementary Fig. S1. Archived SDS-PAGE gel documentation and molecular-mass analysis outputs. (A) Full gel image corresponding to Fig. 2A: lanes 1-3, sucrose density gradient centrifugation; lanes 4-6, ultrafiltration followed by lyophilization; lane 7, molecular-weight marker. (B) Molecular-mass analysis output for panel A.
(C) Full gel image corresponding to Fig. 2B: lanes A-B, ammonium sulfate precipitation; lanes C-D, trichloroacetic acid precipitation; lanes E-F, cesium chloride gradient centrifugation; lane G, molecular-weight marker.(D) Molecular-mass analysis output for panel C.

A

Supplementary Fig. S2. Full uncropped SDS-PAGE gel images and molecular-mass analysis outputs. (A) Full gel image corresponding to Fig. 2A: lanes 1-3, sucrose density gradient centrifugation; lanes 4-6, ultrafiltration followed by lyophilization; lane 7, molecular-weight marker.
